# Synthesis and Characterization of Injectable Hydrogels with Varying Collagen–Chitosan–Thymosin β4 Composition for Myocardial Infarction Therapy

**DOI:** 10.3390/jfb9020033

**Published:** 2018-03-26

**Authors:** Achmad Dzihan Shaghiera, Prihartini Widiyanti, Helmy Yusuf

**Affiliations:** 1 Biomedical Engineering Study Program, Department of Physics, Faculty of Science and Technology, Airlangga University, Surabaya 60115, East Java, Indonesia; achmad.dzihan@gmail.com; 2 Institute of Tropical Disease, Airlangga University, Surabaya 60115, East Java, Indonesia; 3 Department of Clinical Pharmacy, Faculty of Pharmacy, Airlangga University, Surabaya 60286, East Java, Indonesia; helmy.yusuf@gmail.com

**Keywords:** myocardial infarction, hydrogel, collagen, chitosan, thymosin β4

## Abstract

Thirty percent of global mortalities are caused by cardiovascular disease, and 54% of the aforementioned amount is instigated by ischemic heart disease that triggered myocardial infarction. Myocardial infarction is due to blood flow cessation in certain coronary arteries that causes lack of oxygen (ischemia) and stimulates myocardial necrosis. One of the methods to treat myocardial infarction consists in injecting cells or active biomolecules and biomaterials into heart infarction locations. This study aimed to investigate the characteristics of a collagen–chitosan-based hydrogel with variations in its chitosan composition. The prepared hydrogels contained thymosin β4 (Tβ4), a 43-amino acid peptide with angiogenic and cardioprotective properties which can act as a bioactive molecule for the treatment of myocardial infarction. A morphological structure analysis showed that the hydrogels lacked interconnecting pores. All samples were not toxic on the basis of a cytotoxicity test. A histopathological anatomy test showed that the collagen–chitosan–thymosin β4 hydrogels could stimulate angiogenesis and epicardial heart cell migration, as demonstrated by the evaluation of the number of blood vessels and the infiltration extent of myofibroblasts.

## 1. Introduction

Cardiovascular disease is the prime cause of death in the world. In 2008, the World Health Organization (WHO) stated that 17.3 million people died from this particular disease in that year; this represents 30% of mortalities globally, and 54% of that number was caused by ischemic cardiac disease. Heart failure can be caused by many factors, with the most common one being myocardial infarction (MI). Acute myocardial infarction is the cessation of blood flow in certain coronary arteries, causing a lack of oxygen (ischemia) and stimulating myocardial necrosis [1]. MI can be caused by many factors, but the most common one is coronary blockage that disturbs the blood flow. The blockage is instigated by plaque rupture that induces thrombocyte aggregation, forming a thrombus, and coronary spasms [2]. Irreversible damage to the myocardium occurs about 20 min after MI. In the following 3 h, damage spreads to other areas, destroying billions of cells. Within two months, the affected part of the heart infarction is replaced by scar tissue, a process called cardiac remodeling. This contributes to inefficient mechanical blood pumping, which causes congestive heart failure (CHF) to the patient [3,4].

Full heart transplant is the best choice for heart failure patients, but this option is limited by the quantity of ready donors, the complications due to the immune response, and the long-term graft failure potential [5]. There are various developments in biomaterials to implement strategies for heart failure treatment, such as microporous hollow fiber oxygenator membranes, artificial heart valves, coronary artery bypass grafting, stents, etc. [6]. However, these strategies are not efficient enough to halt the progress of myocardial infarction, especially in late-stage heart failure patients [3].

Another alternative is the injection of cells and biomaterials into the heart infarction site. Various types of cell are used, including stem cells, which are obtained from a patient using an autologous graft method, with various results [7]. The obstacle when using the straight injection method is that only a few of the cells will remain in the infarction location. Around 50% of the inserted cells will perish after injection, and only 10% of the cells will survive for a week [8,9]. This might be caused by the low viscosity of the body’s saline solution and by apoptosis when the cells are exposed to the ischemic infarction environment [10]. The healing process can be improved by using delivery media that will allow the cells to remain alive in the infarction area [11].

Hydrogels are one of the potential media useful in cardiac cells treatment. Hydrogels for heart treatment can be prepared by introducing in them stem cells, progenitor cells, and bioactive molecules. Hydrogels offer a good microenvironment for cells or bioactive molecules enabling their survival, and their viscosity can keep the aforementioned cells or molecules in the heart. Hydrogels can also supply cardiac progenitors with a favorable environment for differentiation into cardiomyocytes [12].

Collagen is a biocompatible material. As a component of the extracellular matrix, collagen contributes to tissues’ tensile strength. Collagen is also a component of the pericardium. It plays an important role in cell attachment and proliferation and has high biodegradability [11].

Chitosan is known to have antibacterial properties [13]. Moreover, chitosan has been vastly incorporated in biomaterials because of its hydrophilic, non-toxic, positively charged traits that enhance biomaterials’ compressive strength and other characteristics [14]. Chitosan has the potential to stabilize the heart wall by reducing the dilatation caused by myocardial infarction [15]. Thymosin β4 (Tβ4), a 43-amino acid peptide with angiogenic and cardioprotective effects [16], has been known to help cardiac cells’ migration from adult heart’s explants to repair tissue damages [17].

Chiu (2011) [11] described a collagen–chitosan hydrogel that was synthesized by a mixing method, that is, in the form of a solution to which thymosin β4 was then added in different amounts as a progenitor protein to trigger angiogenesis (using a swelling ratio of 1 < x < 1.25). This was to create a hydrogel morphology with creases and pores, forming interconnections. The present study aimed to synthesize and characterize hydrogels with varying compositions, using type 1 collagen and chitosan in the ratios of 2.5:0.625 mg/mL, 2.5:1.25 mg/mL, 2.5:1.875 mg/mL, and 2.5:2.5 mg/mL. The characterization was carried out using SEM (Scanning Electron Microscope) to identify the morphological structure of the hydrogels. In addition, a cytotoxicity test was performed to examine the degree of the hydrogels’ toxicity, and an anatomical histopathology test was carried out to detect epicardial cell migration and angiogenesis in the presence of the hydrogels.

## 2. Results

### 2.1. Morphology Test

A morphology test was performed using SEM (Inspect™ S50, FEI, Tokyo, Japan) to observe the morphological structure of the hydrogel surface and to determine whether there were creases and interconnected pores in the hydrogel. The test was carried out on samples with a chitosan content of 1.25 mg/mL. The results of the cytotoxicity and the histopathological tests showed that the samples with a chitosan content of 1.25 mg/mL gave optimal results. The morphology test results using a magnification of 500×, 1.000×, and 10.000× were then compared with the SEM results reported by Chiu, et al. (2011) [11].

**Figure 1:**
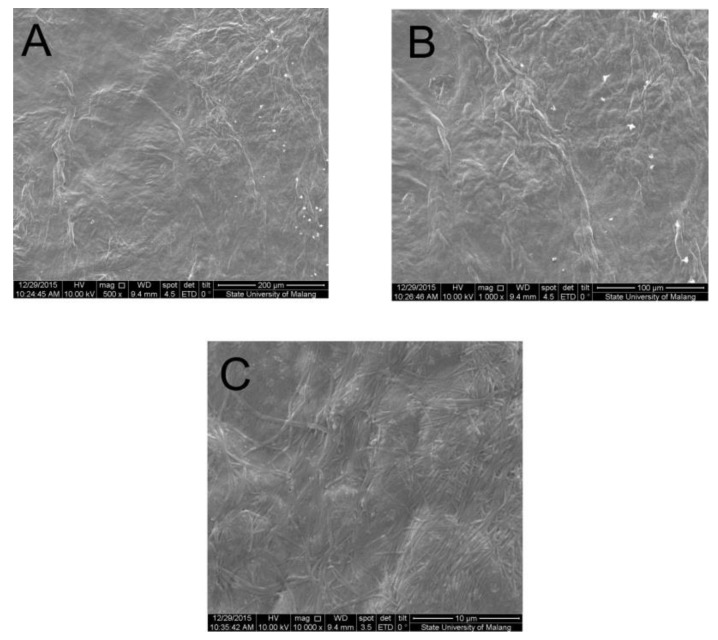
SEM images of a hydrogel sample containing 1.25 mg/mL of chitosan, (**A**) magnified 500×; (**B**) magnified 1.000×; (**C**) magnified 10.000×.

Figure 1 shows the presence of creasing patterns, and the absence of interconnecting pores in the sample containing chitosan at 1.25 mg/mL. This result is different from those of a previous research done by Chiu et al. (2011) [11] that reported the a chitosan–collagen mixture formed clear creasing patterns and many interconnecting pores. The presence of interconnecting pores in the hydrogel structure is very important to reduce thymosin β4 diffusion from of the hydrogel, because the pores act as a diffusion barrier to trap small molecules such as the aforementioned thymosin β4 [11].

### 2.2. Cytotoxicity Test with MTT Assay (3-(4,5-Dimethylthiazol-2-yl)-2,5-diphenyltetrazolium Bromide)

**Figure 2:**
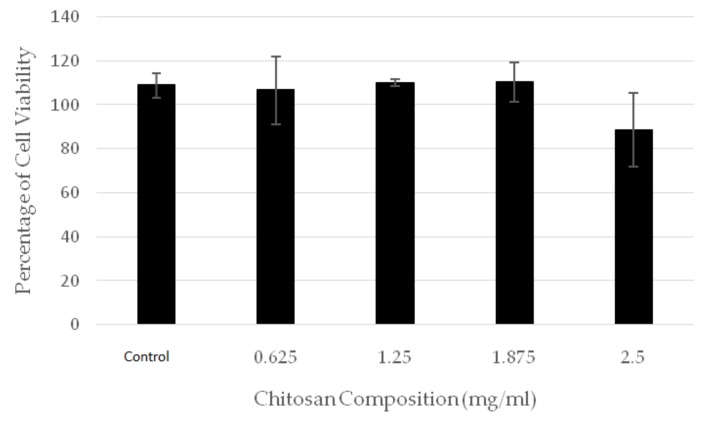
The percentage cell viability in hydrogels with different chitosan contents.

The cytotoxicity test result showed the percentage of surviving cells in five types of hydrogel samples with different chitosan composition, corresponding to 0.625, 1.25, 1.875, and 2.5 mg/mL. The percentage of surviving cells obtained by MTT assays are shown in Figure 2.

The result showed that all hydrogel samples were not toxic, because the percentage of cell viability was above 50% for all samples. Furthermore, almost all the samples (with the exception of the sample with chitosan composition of 2.5 mg/mL) showed proliferation or cell growth, since they displayed a cell viability exceeding 100%.

### 2.3. Anatomy Histopathology

To discover the effect of angiogenesis and epicardial cells migration from the hydrogels into the body, an anatomy histopathology test was conducted in 2–3-months-old Wistar male mice, weighing about 150 g. Before performing the test, the research team passed the animal ethic code test (Ethical Clearance Number: 463-KE) from the Animal Care Use Committee, Faculty of Veterinary, Airlangga University. Five collagen–chitosan hydrogel compositions were tested in the mice as follows: collagen–chitosan 2.5:0.625 mg/mL, 2.5:1.25 mg/mL, 2.5:1.875 mg/mL, 2.5:2.5 mg/mL, and control 2.5:0 mg/mL; thymosin β4 1000 ng was introduced in each variation, as described more clearly in Table 1. A total of 30 male mice were randomly injected, so each group consisted of six mice.

**Figure 3:**
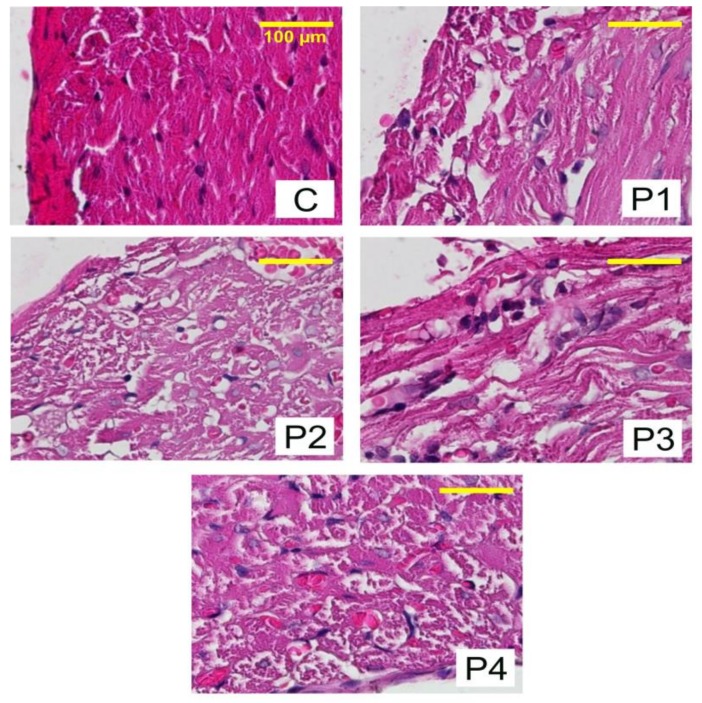
Heart tissue preparations after treatment with hydrogels containing different amounts of chitosan to evaluate angiogenesis and epicardial cell migration. C: 0 mg/mL; P1: 0.625 mg/mL; P2: 1.25 mg/mL; P3: 1.875 mg/mL; P4: 2.5 mg/mL.

The treatment was conducted once in each mouse after a proper adaptation time. A week afterwards, the mice underwent a necropsy in the anatomy pathology laboratorium, Faculty of Veterinary, Airlangga University to obtain heart preparations for subsequent examination. The results of the anatomy histopathology analysis are shown in Figure 3.

**Figure 4:**
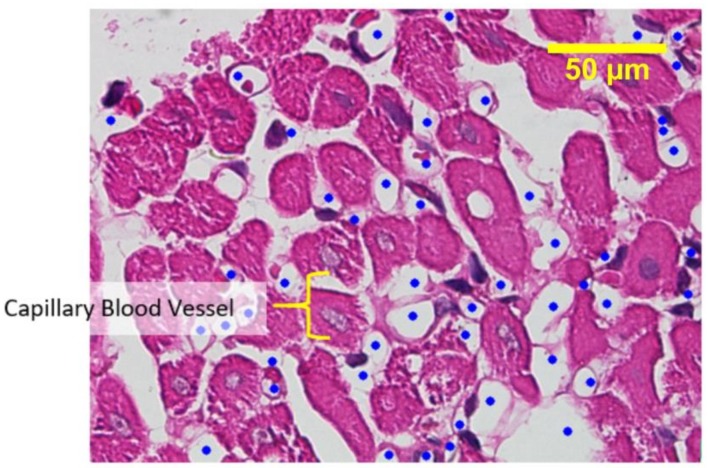
Capillary blood vessels in a sample containing 0.625 mg/mL of chitosan.

**Figure 5:**
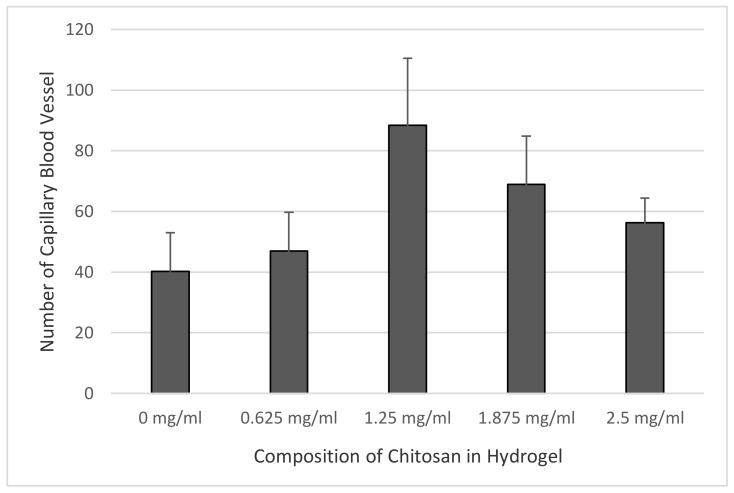
The relationship between chitosan composition and the number of capillary blood vessels in each hydrogel.

Figure 4 shows capillary blood vessels in the preparations. The amount of capillary blood vessels in the epicardium area of each sample was counted through observation under light microscope, and the results are shown in Figure 5. The data were tabulated and analyzed using one-way ANOVA (Analysis of Variance) statistical analysis with a degree of freedom set at 95% (*p* = 0.05), to see any differences from the treatment group. The statistical analysis also included the post-hoc Tukey test, to detect any influence among the groups.

Since the data distributed normally and homogenously, the one-way ANOVA statistical analysis was performed with a degree of freedom of 95%. The results confirmed the hypothesis of a significant change in the growth of capillary blood vessels in all samples, with significance <0.05. All treatment groups showed significant difference with respect to the control group, hence it was established that chitosan significantly influences angiogenesis or the growth of new capillary blood vessels.

The post-hoc Tukey test analysis indicated that not all treatment groups showed a significant difference with respect to the control group. A significant difference was only observed in samples II and III (chitosan composition of 1.25 mg/mL and 1.875 mg/mL). Afterwards, using the same preparations and treatment groups, the activation of myofibroblast cell infiltration from the epicardium to the myocardium was analyzed.

The Kruskal–Wallis test of non-parametrical statistical analysis was conducted because of heterogenous data variance. The mean rank score showed that the mean rank of the sample III group treatment (chitosan composition of 1.875 mg/mL) was higher than that of the other groups. Hence, the Kruskal–Wallis test was performed to confirm the mean rank difference, which resulted statistically significant. The Kruskal–Wallis test indeed confirmed that the hypothesis (the treatment have involved on myofibroblast cells infiltration) was accepted, as the p value was 0.001, indicating that the treatment had significant influence on myofibroblast cells infiltration.

## 3. Discussion

Functional blood vessels are important in the formation and the maintenance of a tissue [18]. Current strategis to stimulate the formation of blood vessels in an ischemic area often involve the injection of endothelial cells or their precursors, or of angiogenic growth factors, such as VEGF [19]. Cell injection interventions are limited by the availability of the type and source of the cells, the reduced success of cell engraftment, and the reduced cell survival post-injection [20]. The ability of vascular cells, with their implants, to survive depends on how fast vascular organization can be stimulated by angiogenic factors, before they are degraded in the implants [18].

It is known that Tβ4 is able to increase the survival ability of vascular cells and cardiomyocytes. It can also induce endogenous endothelial cells to migrate into ischemic areas following myocardial infarction, which stimulates the formation of new blood vessels [21]. From previous research conducted by Smart et al. [22], Tβ4 appears to be more efficient than other angiogenic factors because is able to initiate angiogenesis and induce vascular stability by promoting the recruitment and differentiation of endothelial cells and smooth muscle cells.

Biomolecules, inserted by injection in a liquid form, are very vulnerable to quick degradation, thus requiring repeated injection and dose escalation for therapy [23]. Therefore, media for biomolecules encapsulation are needed to provide biomolecule release and consequent local effects at the sites of implantion.

This research examined the effects of various chitosan compositions of chitosan–collagen hydrogels as control media fort the release of Tβ4 for myocardial infarction therapies, on the basis of in vitro and in vivo tests. Polycation–polyanion complexes formed by collagen and chitosan have previously been studied, especially in drug delivery systems. Gel formation occurs in two stages: first, the formation of collagen fibrils and the spread of chitosan around them and, second, the establishment of electrostatic interactions and hydrogen bonding between the molecules of chitosan and collagen [3]. The addition of NaOH aims to neutralize the acidic solution of collagen I which shrinks the polymer structure of collagen and to favor the coordination of the polyelectrolyte components into the gel [24]. Collagen helps cells to survive, but it has a very rapid degradation. The addition of chitosan can increase the mechanical strength of the hydrogel and reduce the rate of collagen degradation by collagenase enzymes [3]. The chitosan molecule has hydroxyl and amino clusters while collagen has carbonyl and amino clusters [3]. There are two known interaction types taking place between collagen and chitosan while forming a complex. The first one is an electrostatic interaction when a polyanion–polycation complex is formed between two types of polyelectrolytes. The second interaction consists of hydrogen bonds between collagen and chitosan [25]. This cannot be seen from the morphological structure of the samples because of the minimal interconnections of pores, even though the crease patterns are clearly visible. This interaction may be caused by a non-neutral pH in the mixing process, which results in the charge in the collagen not being totally negative. This will affect the formation of polyelectrolyte coordination between collagen and chitosan, as well as the shrinkage of the collagen structure [24].

**Figure 6:**
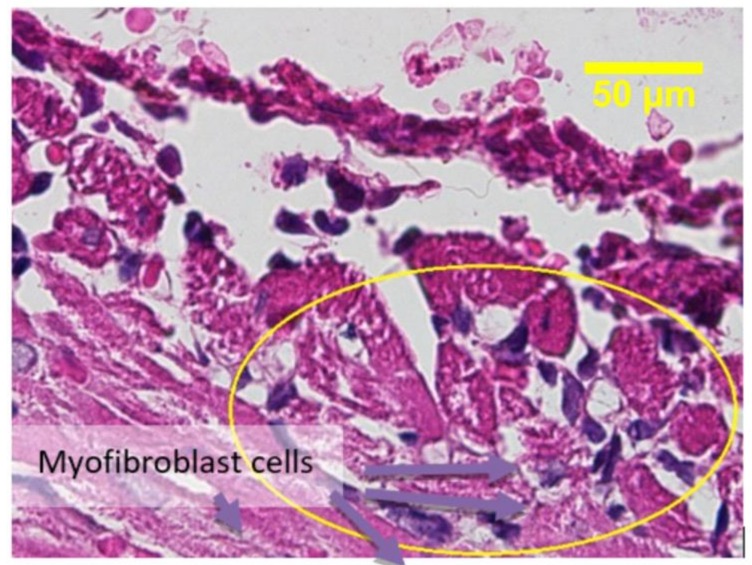
Active myofibroblast cell infiltration in sample 1.5 (chitosan 0.625 mg/mL).

**Figure 7:**
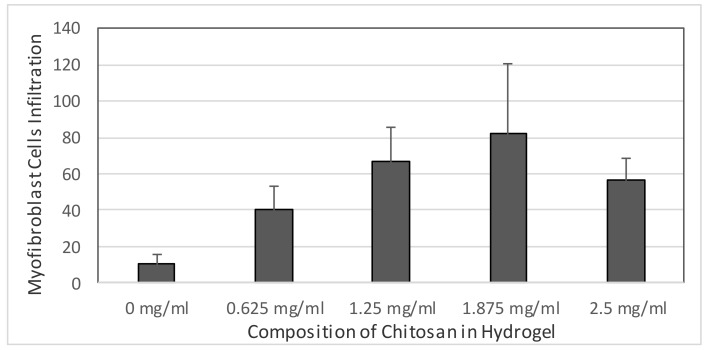
The relationship between the chitosan composition of the hydrogels and myofibroblast cells infiltration.

The results of the anatomy histopathology test displayed in Figure 4 and Figure 5 confirmed that the addition of chitosan resulted in higher and significant amounts of capillary blood vessels formed compared to the addition of the collagen-only hydrogel. The higher amount of capillary displayed signs of angiogenesis. Furthermore, Figure 6 and Figure 7 confirmed that the infiltration of myofibroblast cells was also significantly higher in the presence of chitosan compared to the collagen-only hydrogel. Myofibroblast cell infiltration means that myofibroblast cells, which are initially inactive and located in the epicardium, become active and migrate to the myocardium. This can be caused by two factors. First, the slow release of thymosin β4 from collagen—chitosan hydrogels occurs with a different kinetics release compared to the hydrogel containing collagen only, which releases thymosin β4 within three days in vitro. In an in vivo environment, collagen is very easily degraded enzymatically by collagenases, and this process accelerates the release of thymosin β4 as a consequence of a damaged collagen structure [11]. Second, Deng et al. (2011) [15] argued that the addition of chitosan to a collagen hydrogel can increase the ability of the hydrogel to support the angiogenic progenitor phenotype in vivo. However, the results of the cytotoxicity test, based on cell viability and an anatomical histopathology test as well as on the number of capillary vessels and myofibroblast cell infiltration parameters, showed that the collagen–chitosan sample with a composition ratio of 1:1 resulted in a decline of the pro-angiogenic trend. This is most likely because the matrix formed is denser as the chitosan composition increases. As a result, thymosin β4 release becomes slower and this causes a decline in some of the measured parameters. It is advisable to consider new options related to the type of chitosan taking into account also its antibacterial and antioxidant characteristics. Tamer et al. (2017) modified chitosan to make O-amine-functionalized chitosan, which exhibited good results in comparison with native chitosan in terms of antibacterial and antioxidant properties [13].

## 4. Materials and Methods 

### 4.1. Materials and Equipment

The instruments used in this research were incubator, vortex, magnetic stirrer, microscope, deep freezer, Scanning Electron Microscopy (Inspect™ S50, FEI, Japan), ELISA Reader (Multiscan EX, Thermo Scientific™, Cincinnati, OH, USA), handspun, 10 mL syringe, 24-well plate, micro pipette, test tubes, and beaker. The materials employed were: water soluble chitosan (75–90% degree of deacetylation, MW = 500 kDa, C.V Bio Chitosan, Tangerang, Indonesia), type 1 collagen obtained from rat tail (Gibco^®^, Thermo Scientific™, Cincinnati, OH, USA), PBS solution (Sigma-Aldrich^®^, Queenstown, Singapore), thymosin β4 (Tβ4) (Prospec^®^, Tel Aviv, Israel), distilled water, hydroxide sodium, acetate acid, and Wistar adult white male mice.

### 4.2. Hydrogel Synthesis

The amounts of chitosan in the samples were: 0 (control), 0.625 (I), 1.25 (II), 1.875 (III), and 2.5 mg/mL (IV). The preparation of the samples started with the incorporation of the main ingredients (chitosan, collagen, and thymosin β4 1000 ng) into a microcube, followed by vortexing in order to obtain a homogenous mix. After that, 10× PBS (1/10 of the total volume of solution) and 1N Sodium Hydroxide (0.025× volume of collagen) were added to neutralize the solution. Afterwards, the materials were vortexed and placed on ice for about 3 min, and then gelation was carried out for 1 h in an incubator at a temperature of 36–37 °C; hydrogels were formed at the end of gelation [11]. The sample were characterized by various tests, as described in this section.

### 4.3. Morphology Test

The SEM test was done to analyze the morphological structure of the hydrogels. Each hydrogel cell was dried first before being analyzed by SEM. The drying process was done in an oven for several minutes, and then the sample was fixed onto the microscope holder that had been coated with double-side carbon tape. The sample was then coated with a mixture of Au–PD (gold and palladium) by a sputter coater (SC7620, Quorum Technologies, East Sussex, UK). Lastly, the sample was analyzed by SEM, recording the results obtained through 500× and 1000× magnification [26].

### 4.4. Cytotoxicity Test with MTT Assay

The test was done to determine whether the synthesized hydrogel was cytotoxic. Monolayers of BHK-21 cultured cell grown in the incubator for 48 h were used. The media was eliminated, and the cells were washed with PBS (Phosphate Buffered Saline) to arrest cell metabolism and remove serum traces. Then, the cell culture was trypsinized with versene–trypsin 0.25% to prevent cells from forming colonies and to collect all cells attached to the roux culture bottle walls [27].

The cells in 100 µL Eagle medium were plated into 96-microwell plates. A precise volume of 25 µL of the MTT (3-(4,5-Dimethylthiazol-2-yl)-2,5-diphenyltetrazolium bromide) reagent was added in each well. The cell culture was incubated for 4 h at the temperature of 37 °C, then DMSO (Dimethyl Sulfoxide), in the amount of 50 µL, was added in each well to stop the reaction, and the plate was vortexed for 5 min to evenly combine DMSO. The formation of formazan was determined with an ELISA Reader (Multiscan EX, Thermo Scientific™, Cincinnati, OH, USA) at 630 nm wave length [27].

The number of surviving cells was determined on the basis of: membrane damage parameters, synthesis disturbance, macromolecular degradation, metabolism modification, and cell morphology changes. The toxicity level was determined on the basis of the extent of damage to the cell membrane indicated by the cell uptake of a coloring agent, such as trypan blue. The final result of the toxicity test indicated the percentage of surviving cells. According to Spielmann (2007), a tested compound is defined as non-toxic if the percentage of surviving cells is over 50% [28].

### 4.5. Anatomy Histopathology Test 

To find out the in vivo effect on angiogenesis of the injected hydrogels, various tests were performed, among which an anatomy histopathology analysis. The hydrogels were tested in 2–3-months-old Wistar male mice, weighing about 150 gm. The collagen–chitosan hydrogels of varying compositions (2.5:0.625 mg/mL, 2.5:1.25 mg/mL, 2.5:1.875 mg/mL, 2.5:2.5 mg/mL, and 2.5:0 mg/mL, the last-mentioned as a control), all containing thymosin β4 (1000 ng), were injected subcutaneously in the upper back of the mouse using a 23 ¾ G syringe. Six mice from each group (including the control mice) were necropsied on the seventh day after the injection for the histopathology test. The samples were mixed in a neutral buffered formaline (NBF) solution 10%, dehydrated with various concentration of alcohol (70%, 80%, 90%, and 96%), cleared with xylol, and embedded in paraffin. Once solidified, the samples were cut using a microtome at a 5 µ thickness. Finally, the samples were rehydrated and colored with hematoxylin and eosin [11]. The examination and interpretation of the samples were performed by the veterinary pathology laboratory of the Veterinarian Faculty, Airlangga University.

## 5. Conclusions

In this research, we developed an injectable collagen—chitosan hydrogel with several variations of chitosan composition to observe the release of thymosin β4 and the consequent capillary vessel growth and myofibroblast cell migration. Generally, it can be concluded that the higher the chitosan content in the hydrogel, the higher the number of capillary vessels in the heart and the more extensive the migration of myofibroblasts. Moreover, the collagen–chitosan hydrogel can be considered as a *carrier* of other negatively charged active biomolecules because of its capabilities and thus presents various applications.

## Figures and Tables

**Table 1:** Treatment Group.

Materials	Control	Sample 1	Sample 2	Sample 3	Sample 4
Collagen	2.5 mg/mL	2.5 mg/mL	2.5 mg/mL	2.5 mg/mL	2.5 mg/mL
Chitosan	0 mg/mL	0.625 mg/mL	1.25 mg/mL	1.875 mg/mL	2.5 mg/mL
Thymosin β4	1000 ng	1000 ng	1000 ng	1000 ng	1000 ng

## References

[1] Marelli T.M. (2008). Buku Saku Dokumentasi Keperawatan.

[2] Thygesen K., Alpert J.S., White H.D. (2007). Universal definition of myocardial infarction. Eur. Heart J..

[3] Chen Q.-Z., Harding S.E., Ali N.N., Lyon A.R., Boccaccini A.R. (2008). Biomaterials in cardiac tissue engineering: Ten years of research survey. Mater. Sci. Eng. R Rep..

[4] Radhakrishnan J., Krishnan U.M., Sethuraman S. (2014). Hydrogel based injectable scaffolds for cardiac tissue regeneration. Biotechnol. Adv..

[5] Jameel M.N., Zhang J. (2009). Heart failure management: The present and the future. Antioxid. Redox Signal..

[6] Nelson D.M., Ma Z., Fujimoto K.L., Hashizume R., Wagner W.R. (2011). Intra-myocardial biomaterial injection therapy in the treatment of heart failure: Materials, outcomes and challenges. Acta Biomater..

[7] Ye Z., Zhou Y., Cai H., Tan W. (2011). Myocardial regeneration: Roles of stem cells and hydrogels. Adv. Drug Deliv. Rev..

[8] Müller-Ehmsen J., Whittaker P., Kloner R.A., Dow J.S., Sakoda T., Long T.I., Laird P.W., Kedes L. (2002). Survival and development of neonatal rat cardiomyocytes transplanted into adult myocardium. J. Mol. Cell. Cardiol..

[9] Zhang M., Methot D., Poppa V., Fujio Y., Walsh K., Murry C.E. (2001). Cardiomyocyte grafting for cardiac repair: Graft cell death and anti-death strategies. J. Mol. Cell. Cardiol..

[10] Christman K.L., Fok H.H., Sievers R.E., Fang Q., Lee R.J. (2004). Fibrin glue alone and skeletal myoblasts in a fibrin scaffold preserve cardiac function after myocardial infarction. Tissue Eng..

[11] Chiu L.L., Radisic M. (2011). Controlled release of thymosin beta4 using collagen-chitosan composite hydrogels promotes epicardial cell migration and angiogenesis. J. Control. Release.

[12] Giraud M.N., Tevaearai H., Boccaccini A.R., Harding S.E. (2011). Tissue Engineering Approaches for Myocardial Bandage: Focus on Hydrogel Constructs. Myocardial Tissue Engineering.

[13] Tamer T.M., Hassan M.A., Omer A.M., Valachová K., Eldin M.S.M., Collins M.N., Šoltés L. (2017). Antibacterial and antioxidative activity of *O*-amine functionalized chitosan. Carbohydr. Polym..

[14] Fukuda J., Khademhosseini A., Yeo Y., Yang X., Yeh J., Eng G., Blumling J., Wang C.F., Kohane D.S., Langer R. (2006). Micromolding of photocrosslinkable chitosan hydrogel for spheroid microarray and co-cultures. Biomaterials.

[15] Deng C., Zhang P., Vulesevic B., Kuraitis D., Li F., Yang A.F., Griffith M., Ruel M., Suuronen E.J. (2010). A collagen-chitosan hydrogel for endothelial differentiation and angiogenesis. Tissue Eng. Part A.

[16] Jo J.O., Kim S.R., Bae M.K., Kang Y.J., Ock M.S., Kleinman H.K., Cha H.J. (2010). Thymosin beta4 induces the expression of vascular endothelial growth factor (VEGF) in a hypoxia-inducible factor (HIF)-1alpha-dependent manner. Biochim. Biophys. Acta.

[17] Bock-Marquette I., Saxena A., White M.D., Dimaio J.M., Srivastava D. (2004). Thymosin beta4 activates integrin-linked kinase and promotes cardiac cell migration, survival and cardiac repair. Nature.

[18] Kraehenbuehl T.P., Ferreira L.S., Zammaretti P., Hubbell J.A., Langer R. (2009). Cell-responsive hydrogel for encapsulation of vascular cells. Biomaterials.

[19] Ferrara N., Alitalo K. (1999). Clinical applications of angiogenic growth factors and their inhibitors. Nat. Med..

[20] Mooney D.J., Vandenburgh H. (2008). Cell delivery mechanisms for tissue repair. Cell Stem Cell.

[21] Smart N., Risebro C.A., Melville A.A., Moses K., Schwartz R.J., Chien K.R., Riley P.R. (2007). Thymosin beta4 induces adult epicardial progenitor mobilization and neovascularization. Nature.

[22] Smart N., Risebro C.A., Clark J.E., Ehler E., Miquerol L., Rossdeutsch A., Marber M.S., Riley P.R. (2010). Thymosin beta4 facilitates epicardial neovascularization of the injured adult heart. Ann. N. Y. Acad. Sci..

[23] Lin C.-C., Metters A.T. (2006). Hydrogels in controlled release formulations: Network design and mathematical modeling. Adv. Drug Deliv. Rev..

[24] Maeda H., Sano A., Fujioka K. (2004). Controlled release of rhBMP-2 from collagen minipellet and the relationship between release profile and ectopic bone formation. Int. J. Pharm..

[25] Taravel M.N., Domard A. (1996). Collagen and its interactions with chitosan: III. Some biological and mechanical properties. Biomaterials.

[26] Liu F., Wu J., Chen K., Xue D. (2010). Morphology Study by Using Scanning Electron Microscopy. Microsc. Sci. Technol. Appl. Educ..

[27] Riss T., Moravec R., Niles A.L., Duellman S., Benink H.A., Worzella T.J., Minor L., Sittampalam G., Coussens N., Brimacombe K., Al E. (2013). Cell Viability Assays. Assay Guidance Manual.

[28] Spielmann H., Hoffmann S., Liebsch M., Botham P., Fentem J.H., Eskes C., Roguet R., Cotovio J., Cole T., Worth A. (2007). The ECVAM international validation study on in vitro tests for acute skin irritation: Report on the validity of the EPISKIN and EpiDerm assays and on the Skin Integrity Function Test. Altern. Lab. Anim..

